# *Plasmodium berghei* bio-burden correlates with parasite lactate dehydrogenase: application to murine *Plasmodium* diagnostics

**DOI:** 10.1186/s12936-015-1027-2

**Published:** 2016-01-04

**Authors:** Sai Lata De, Danielle I. Stanisic, Fabian Rivera, Michael R. Batzloff, Christian Engwerda, Michael F. Good

**Affiliations:** Institute for Glycomics, Griffith University, Gold Coast, QLD Australia; QIMR Berghofer Medical Research Institute, Brisbane, QLD Australia

**Keywords:** Malaria, pLDH, Murine *Plasmodium*, Parasite bio-burden

## Abstract

**Background:**

The spectrum of techniques to detect malaria parasites in whole blood is limited to measuring parasites in circulation. One approach that is currently used to enumerate total parasite bio-burden involves the use of bio-luminescent parasites. As an alternative approach, this study describes the use of a commercial ELISA human parasite lactate dehydrogenase (pLDH) detection kit to estimate total parasite bio-burden in murine malaria models.

**Methods:**

The cross reactivity of pLDH in a commercial human malaria pLDH diagnostic kit was established in different components of blood for different murine malaria models. The use of pLDH as a measure of parasite bio-burden was evaluated by examining pLDH in relation to peripheral blood parasitaemia as determined by microscopy and calculating total parasite bio-burden using a bio-luminescent *Plasmodium berghei* ANKA luciferase parasite.

**Results:**

The pLDH antigen was detected in all four murine *Plasmodium* species and in all components of *Plasmodium*-infected blood. A significant correlation (*r* = 0.6922, *P* value <0.0001) was observed between total parasite bio-burden, measured as log average radiance, and concentration of pLDH units.

**Conclusions:**

This high throughput assay is a suitable measure of total parasite bio-burden in murine malaria infections. Unlike existing methods, it permits the estimation of both circulating and sequestered parasites, allowing a more accurate assessment of parasite bio-burden.

## Background

Malaria continues to be a leading cause of mortality in endemic countries, despite control and eradication initiatives, such as the use of insecticide-treated bed nets and anti-malaria drug treatment [1]. Clinical disease typically presents as fever, chills, severe headache, and vomiting, and requires a prompt diagnosis followed by effective treatment to ensure resolution of the infection. Importantly, disease outcomes are directly correlated with total parasite burden [2]. In rodent malaria models, Giemsa-stained blood films (the gold-standard microscopy technique) [3, 4], PCR [5], flow cytometry [6, 7], fluorescent [8, 9] or DNA/RNA [10–12] markers and radioisotope labelling [11] have been used to measure parasitaemia in in vivo preclinical efficacy studies for anti-malaria drugs and vaccine candidates. However, these approaches are limited to detecting circulating parasites and do not account for sequestered parasites, which is important for determining total parasite bio-burden. Bio-luminescence imaging, using transgenic parasite that express the firefly luciferase reporter protein, has been used not only to measure parasite growth in vivo and in vitro [10], but also to visualize in vivo the parasite sequestration in deep tissues [13–16].

Various assays using specific antibodies to parasite antigens have been employed [17–24]. Immunochromatographic assays, which utilize monoclonal antibodies against specific *Plasmodium* enzymes, are under development. The most promising antigens explored so far include: histidine rich protein-2 (HRP-2) [25], parasite-specific lactate dehydrogenase (pLDH) [18, 19, 26–28], and aldolase [29, 30]. These enzymes are involved in metabolic pathways essential for the growth and survival of *Plasmodium* parasites [29].

The enzyme pLDH is a soluble, energy-producing enzyme that is involved in the last step of the glycolytic pathway [29]. As the red blood cells do not have functional mitochondria and the parasites have minimum oxygen uptake for the citric acid cycle [31], it is highly dependent on anaerobic glucose metabolism [32, 33]. pLDH is produced by both asexual blood-stage parasites as well as the sexual stages, with a larger quantity of pLDH being produced during the asexual stage [29]. pLDH antigen is preferable as a diagnostic marker over other antigens such as HRP-2, which is limited to *Plasmodium falciparum* only [34]. Moreover, some *P. falciparum* strains have a deletion in the HRP-2 gene, resulting in false negative tests [35]. Unlike HRP-2, pLDH does not persist in the blood [36, 37] and is cleared immediately post-active infection [18–20, 22, 38, 39], thus making pLDH an ideal marker to estimate parasite bio-burden at the time of the assay.

Previously, monoclonal antibodies specific for pLDH have been used to determine the sensitivity of *Plasmodium berghei* to anti-malarial drugs in vitro [40]. A chromogenic pLDH assay has also been employed to enumerate the parasites in the blood of mice challenged with *Plasmodiumyoelii* 17XNL post vaccination with MSP1-19 [41]. However, none of these approaches was compared to an established assay to quantify and validate total parasite bio-burden.

The pLDH amino acid sequence has a 90 % sequence identity amongst all human *Plasmodium* species [33, 42]. For human parasites, monoclonal antibodies against the shared common epitopes can be used to detect all species [43, 44]. Genetic conservation and variation of pLDH across different human and rodent species and strains of *Plasmodium* was reported by Talman et al. [45]. Nucleotide BLAST analysis using 951 nucleotides of the *P. falciparum* 3D7 (*Pf* LDH) gene coding sequence [Accession ID XM_001349953.1] as the reference revealed the following per cent identity in different species of murine *Plasmodium:* 86 % identity with *P. yoelii* 17XNL [Accession ID XM_719008.1]; 85 % with *Plasmodium chabaudi chabaudi* [Accession ID XM_740087.1]; 85 % with *P. berghei* ANKA [Accession ID XM_674309.1]; and 83 % with *Plasmodium vinckei* [Accession ID XM_008624100.1]. The high degree of sequence similarity could potentially be exploited for use in diagnostics for rodent malaria parasites (Table 1).

**Table 1:** pLDH protein sequence alignment analysis of different species of murine *Plasmodium*

pLDH sequence alignment using Clustal Omega		
	1	60
*P. falciparum* 3D7	MAPKAKIVLVGSGMIGGVMATLIVQKNLGDVVLFDIVKNMPHGKALDTSHTNVMAYSNCK	
*P. vinckei*	M **PQRP** KIVLVGSGMIGGVMATLIVQKNLGDVV **M** FDIVK **D** MPHGKALDTSHTNVMAYSNCK	
*P. chabaudi chabaudi*	MAPKAKIVLVGSGMIGGVMATLIVQKNLGDVV **M** FDIVK **D** MPHGKALDTSHTNVMAYSNC **Q**	
*P. yoelii* 17XNL	MAPKAKIVLVGSGMIGGVMATLIVQKNLGDVVLFDIVKNMPHGKALDTSHTNVMAYSNCK	
*P. berghei* ANKA	MAPKAKIVLVGSGMIGGVMATLIVQKNLGDVV **M** FDIVKNMPHGKALDTSHTNVMAYSNCK	
	61	120
*P. falciparum* 3D7	VSGSNTYDDLAGADVVIVTAGFTKAPGKSDKEWNRDDLLPLNNKIMIEIGGHIKKNCPNA	
*P. vinckei*	V **T** GSN **S** YDDL **K** GADVVIVTAGFTK **V** PGKS **E** KEWNRDDLLPLNNKIMIEIGGHIKK **Q** CPNA	
*P. chabaudi chabaudi*	VSGSNTYDDL **K** GADVVIVTAGFTKAPGKSDKEWNRDDLLPLNNKIMIEIGGHIKK **H** CP **H** A	
*P. yoelii* 17XNL	VSGSNTYDDL **KD** ADVVIVTAGFTKAPGKSDKEWNRDDLLPLNNKIMIEIGGHIK **N** NCPNA	
*P. berghei* ANKA	VSGSNTYDDL **KD** ADVVIVTAGFTKAPGKSDKEWNRDDLLPLNNKIMIEIGGHIK **N** NCPNA	
	121	180
*P. falciparum* 3D7	FIIVVTNPVDVMVQLLHQHSGVPKNKIIGLGGVLDTSRLKYYISQKLNVCPRDVNAHIVG	
*P. vinckei*	FIIVVTNPVDVMVQLLHQHSGVPKNKI **V** GLGGVLD **S** SR **F** KYYI **AE** KLNVCPRDVNAHIVG	
*P. chabaudi chabaudi*	FIIVVTNPVDVMVQLLHQHSGVPKNKI **V** GLGGVLDTSRLKYYISQKLNVCPRDVNAHIVG	
*P. yoelii* 17XNL	FIIVVTNPVDVMVQLLHQHSGVPKNKI **V** GLGGVLDTSRLKYYISQKLNVCPRDVNAHIVG	
*P. berghei* ANKA	FIIVVTNPVDVMVQLLHQHSGVPKNKI **V** GLGGVLDTSRLKYYISQKLNVCPRDVNAHIVG	
	181	240
*P. falciparum* 3D7	AHGNKMVLLKRYITVGGIPLQEFINNKLISDAELEAIFDRTVNTALEIVNLHASPYVAPA	
*P. vinckei*	AHGNKMVLLKRYITVGGIPLQEFINN **NK** I **TED** EL **K** A **MA** DRT **I** NTALEIVNLHASPYVAPA	
*P. chabaudi chabaudi*	AHGNKMVLLKRYITVGGIP **I** QEFINNK **K** ISD **QD** LEAIFDRT **I** NTALEIVNLHASPYVAPA	
*P. yoelii* 17XNL	AHGNKMVLLKRYITVGGIPLQEFINNK **K** I **T** D **Q** EL **D** AIFDRTVNTALEIVNLHASPYVAPA	
*P. berghei* ANKA	AHGNKMVLLKRYITVGGIPLQEFINNK **K** I **T** D **Q** EL **D** AIFDRT **I** NTALEIVNLHASPYVAPA	
	241	300
*P. falciparum* 3D7	AAIIEMAESYLKDLKKVLICSTLLEGQYGHSDIFGGTPVVLGANGVEQVIELQLNSEEKA	
*P. vinckei*	AAIIEMAESYLKDL **R** KVLICSTLLEG **E** YGH **K** DIF **A** GTP **L** V **I** G **G** NGVEQVIELQLN **ET** EK **M**	
*P. chabaudi chabaudi*	AAIIEMAESY **IR** DL **R** KVLICSTLLEGQYGH **K** DIF **A** GTP **L** V **I** G **G** NGVEQVIELQLN **AD** EK **K**	
*P. yoelii* 17XNL	AAIIEMAESY **IR** DL **R** KVLICSTLLEGQYGH **K** DIF **A** GTP **L** V **I** G **G** NGVEQVIELQLN **AD** EK **K**	
*P. berghei* ANKA	AAIIEMAESY **IR** DL **R** KVLICSTLLEGQYGH **K** DIF **A** GTP **L** V **I** G **G** NGVEQVIELQLN **AD** EK **K**	
	301	316
*P. falciparum* 3D7	KFDEAIAETKRMKALA	
*P. vinckei*	**H** FD **N** A **V** AET **A** RMKAL **I**	
*P. chabaudi chabaudi*	KFDEA **V** AET **S** RMKAL **V**	
*P. yoelii* 17XNL	KFDEA **V** AET **S** RMKAL **I**	
*P. berghei* ANKA	KFDEA **V** AET **S** RMKAL **I**	

*P. falciparum* 3D7 (*Pf* LDH) [Accession ID XM_001349953.1] is the reference sequence. The bold letters indicate the dissimilarities in the amino acid sequences as compared to the pLDH amino acid sequence of *P. falciparum*. *Plasmodium vinckei* LDH [Accession ID XM_008624100.1]; *P. chabaudi chabaudi* LDH [Accession ID XM_740087.1]; *P. yoelii* 17XNL [Accession ID XM_719008.1]; *P. berghei* ANKA LDH [Accession ID XM_674309.1]

This study investigated the use of a commercial human *Plasmodium* pLDH ELISA diagnostic kit for detecting pLDH antigen as a measure of parasite bio-burden during murine malaria infections. This assay could be established as an alternative approach to measure parasite bio-burden in efficacy studies.

## Methods

### Mice and ethics statement

Female BALB/c mice aged 4–6 weeks were purchased from the Animal Resource Centre (ARC) (Canning Vale, Perth, Australia) and maintained under appropriate ARC and Griffith University conditions. This study was carried out in strict accordance with the National Health and Medical Research Council of Australia guidelines, as detailed in the document, *Australian Code of Practice for the Care and Use of Animals for Scientific Purposes*, *8th ed* [46]. The Griffith University Animal Ethics Committee (GLY/05/12/AEC) and the QIMR Berghofer Medical Research Institute Ethics Committee (A02633M) approved the relevant animal procedures and protocols.

### Parasites and infections

Cloned lines of *P. chabaudi AS*, *P. yoelii 17XNL*, *P. yoelii YM*, *P. berghei* and *P. vinckei* were used (provided by Richard Carter, University of Edinburgh, UK). Stabilates were maintained by intra-venous (IV) and intra-peritoneal (IP) passaging of 10^6^ parasitized red blood cells (pRBC) into naïve BALB/c mice. *Plasmodium berghei* ANKA luc lines (provided by Chris Janse, Leiden University Medical Centre, The Netherlands) were used in the bio-luminescent experiments for in vivo imaging after one in vivo passage in mice.

### Evaluation of parasitaemia by microscopy

Thin blood smears were prepared, air dried, fixed in methanol and stained with Giemsa. Slides were examined using bright field microscopy and counts were performed with 100× magnification. For negative or low parasitaemia films (<1 %), at least 20 fields on the slide were counted. For high parasitaemia, at least 500 RBCs were counted. Percent parasitaemia was calculated as the number of pRBCs multiplied by 100 divided by the number of total RBCs.

### Assessment of pLDH antigen production in murine malaria models

Qualitative and quantitative levels of pLDH antigens in BALB/c mice infected with *P. chabaudi* AS, *P. yoelii* 17XNL, *P. yoelii* YM, *P. berghei* ANKA, and *P. vinckei* were determined using the pLDH SD Malaria Antigen ELISA kit (Standard Diagnostics), a sandwich ELISA for the qualitative detection of human *Plasmodium* pLDH. For quantitative assessment, blood was collected on the following days for each of the rodent malaria parasites: *P. chabaudi* AS: Day 7 (29 %), *P. berghei* ANKA: Day 4 (34 %), *P. vinckei*: Day 4 (32 %), and *P. yoelii* YM: Day 5 (24 %). Recombinant pLDH supplied as the positive control in the kit was used to generate a standard curve dilution series with a starting concentration of 1320 ng/mL in PBS or naïve mouse blood. Fifty microlitres of the samples (negative and positive control, standard curve and test samples) were tested in triplicate and the manufacturer’s instructions were followed. Plates were read on an xMark microplate spectrophotometer (Biorad) at 450 nm, with a reference wavelength of 620 nm. The reading was completed within 30 min of the stop solution being added. The linear range was then logarithmically plotted to generate a linear equation to quantify levels of pLDH units in test samples. Positive wells were used to measure the concentration of pLDH unit, with the cut-off for positivity calculated as the mean + three standard deviations of the wells containing naïve mouse blood or PBS alone. The mean optical densities (ODs) were calculated for standards and used to plot a standard curve OD vs ng/mL recombinant pLDH antigen. The concentration of pLDH units in the samples was calculated from the optical density reading from the linear range of the standard curve (as in Fig. 2) such that 1 unit/mL in the test sample was equivalent to 1 ng/mL.

### Visualization and quantification of luciferase activity in mice infected with *Plasmodium berghei* ANKA luciferase

Luciferase activity in *P. berghei* ANKA luc infected mice was visualized using an intensified-charge-coupled device (I-CCD) video camera of the in vivo Imaging System (IVIS 100, Xenogen) [8]. Mice were anaesthetized using the isofluorane anaesthesia system (XGI-8, Xenogen). Following this, mice were injected IP with d-luciferin dissolved in PBS (100 mg/kg of body weight; Synchem, Kassel, Germany) and measurements were performed within 5 min of injection. Bio-luminescence imaging was acquired with an exposure time of 60 s. Imaging data were analysed using the living image (Xenogen) program. In asynchronous infections, measurements were performed on different days at the same time.

### Statistical analysis

**Fig. 1:**
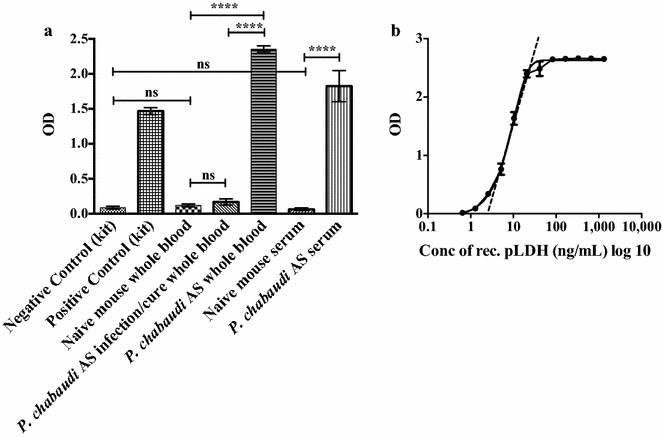
**a** Qualitative detection of pLDH units in different blood components (undiluted) from a mouse infected with *Plasmodium chabaudi AS. Individual bars* represent negative and positive control from the kit, whole blood obtained from a naïve or *P. chabaudi* AS-infected BALB/c mouse, serum obtained from a naïve or *P. chabaudi* AS-infected BALB/c mouse or whole blood obtained from a *P. chabaudi* AS-infected mouse which had been drug treated. **b** Representation of a non-linear standard curve to quantify pLDH units (ng/mL). Recombinant pLDH from the kit was serially diluted in both PBS and blood and plotted as log 10 concentration of recombinant pLDH vs the OD. The *dotted line* shows the linear range of the curve. Each sample was tested in triplicate. *Error bars* represent the mean ± SE of experimental replicates. ****P value <0.0001, ns not significant. One-way ANOVA was used to determine the statistical significance between groups

**Fig. 2:**
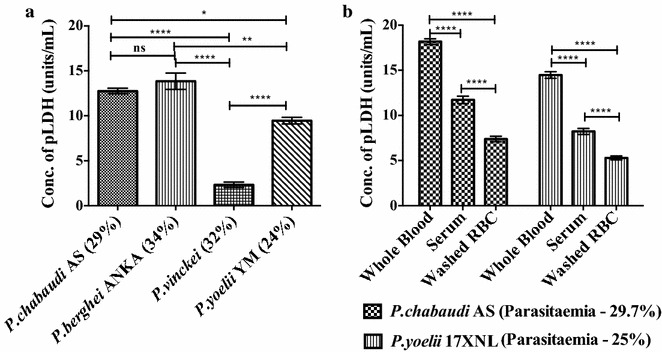
**a** Quantitative detection of pLDH units in whole blood of BALB/c mice infected with different murine *Plasmodium* species and **b** the mean concentration of pLDH units in different components of blood from BALB/c mice infected with *Plasmodium* *chabaudi* AS (29.7 %) and *Plasmodium* *yoelii* 17XNL (25 %). *Percentages* indicate the parasitaemia of the mice as determined by microscopy. Each sample was tested in triplicate. *Error bars* represent mean ± SE of experimental replicates. ****P value <0.0001, **P value <0.01, *P value <0.1, ns not significant. One-way ANOVA was used to determine the statistically significant difference between groups

Graphical and statistical analysis was performed using GraphPad Prism 6. All the datasets are presented as mean ± SE, unless stated otherwise. Non-linear standard curves were generated and the linear part of the curve was used for re-plotting three or more points in logarithmic scale. Logarithmic and arithmetic curves were both used for fitting a trend-line and obtaining an equation, which was used to calculate the concentration of pLDH units from the mean absorbance, for each test sample as described above. The limit of detection (LOD) was calculated as the lowest value in the dilution series that was significantly different from the negative control values. An ANOVA was performed to estimate the LOD. One-way ANOVA was used for the analyses presented in Figs. 1a and 2a, b. Correlation analyses between concentration of pLDH units, parasitaemia and log average radiance were determined using Pearson’s correlation analysis. A Pearson *r* value greater than 0.6 was considered as a strong correlation and shown in the appropriate figures. *P* values less than 0.05 were deemed significant for all statistical analyses.

## Results

### Qualitative detection of pLDH in different blood components in mice infected with *Plasmodium chabaudi* AS

Due to the sequence similarity between the mouse and human *Plasmodium* LDH genes [45], this study utilized the human *Plasmodium* pLDH ELISA kit to assess its ability to detect murine *Plasmodium* pLDH. In a pilot study, pLDH was detected in both whole blood and serum obtained from a *P*. *chabaudi* AS infected mouse on day 7 post infection at 31 % parasitaemia (*P* value <0.0001) (Fig. 1a). Whole blood and serum from both the naïve mouse and a mouse infected with *P. chabaudi* AS and drug cured (100 µL of malarone orally for 5 days), did not have significant detectable pLDH units (Fig. 1a).

### Generation of standard curves using recombinant pLDH

Standard curves were generated for quantitative assessment of pLDH units in infected mice. Using the positive control (rec pLDH) from the commercial ELISA kit, serial dilutions were performed in both PBS and naïve mouse blood to establish standard curves. A representative standard curve from which the linear range was derived is shown in Fig. 1b. The linear range was approximately between 1.18 and 52.8 ng/mL.

### Quantification of pLDH units in different species of murine *Plasmodium*

It was next established if this assay could be used to quantify pLDH units in the whole blood from mice infected with each of the four species of murine malaria. pLDH was detectable in whole blood obtained from mice infected with each of the rodent *Plasmodium* species (Fig. 2a). Despite a similar parasitaemia (24–34 %), pLDH units were significantly lower in mice infected with *P. vinckei* compared to the mice infected with *P. chabaudi* AS, *P. berghei* ANKA and *P. yoelii* YM.

### Quantification of pLDH units in different parasitized blood components

It was then asked whether pLDH was limited to the red blood cell compartment or present both in the serum and pRBC. Blood samples obtained from mice infected with *P*. *chabaudi* AS and *P*. *yoelii* YM were separated into their components and the levels of pLDH units were determined in whole blood, serum and washed RBCs. Washed RBCs were prepared by washing whole blood thrice with PBS by centrifugation at 1500 rpm for 10 min. pLDH was detected in all three sample types, with whole blood showing higher levels of pLDH units compared to the serum and washed red blood cells (Fig. 2b).

### Sensitivity of the pLDH assay in mice infected with *Plasmodiumchabaudi* AS and *Plasmodium berghei* ANKA

**Fig. 3:**
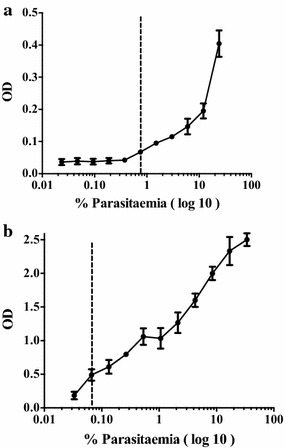
Limit of detection (LOD) of pLDH in BALB/c mice infected with **a** *Plasmodium* *chabaudi* AS (24 % parasitaemia) and **b** *Plasmodium* *berghei* ANKA (34 % parasitaemia). For this assay, infected whole blood was serially diluted in naïve mouse blood. ANOVA was used to estimate LOD. Each sample was tested in triplicate. *Error bars* represent mean ± SE of experimental replicates. The *dotted line* shows the limit of detection

*Plasmodium chabaudi* AS and *P. berghei* ANKA were selected for further studies. The limit of detection (LODs) were established by serially diluting whole parasitized blood in naïve mouse blood. The LOD was approximately 0.7 % parasitaemia for *P. chabaudi* AS and 0.05 % for *P. berghei* ANKA (Fig. 3).

### Relationship between pLDH antigen levels and parasitaemia

**Fig. 4:**
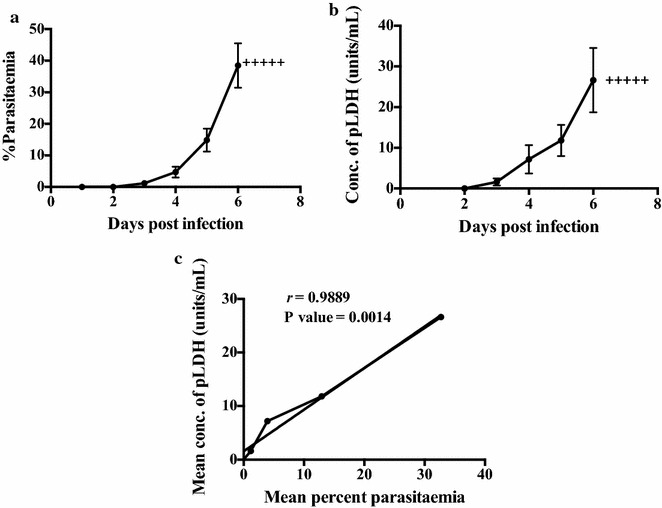
Correlation between the concentration of pLDH units and peripheral blood parasitaemia of BALB/c mice (n = 20) infected with *Plasmodium* *chabaudi* AS. **a** Mean parasitaemia in *P. chabaudi* AS infected mice. **b** Mean concentration of pLDH units in whole blood obtained from *P. chabaudi* AS infected mice. **c** Correlation between the mean concentration of pLDH and peripheral blood parasitaemia (*r* = 0.9889). *Plus* *symbol* indicates that the mice were culled. *Error bars* represent mean ± SE of biological replicates

The correlation between levels of pLDH units and peripheral blood parasitaemias during the course of infection was then assessed. Blood was obtained from mice every day post onset of patent parasitaemia. To ensure that the *P. chabaudi*-infected mice were not anaemic and to allow collection of a suitable volume of blood, a large group of mice (n = 20) were infected and divided into two cohorts. Blood from each cohort was collected on alternate days. A strong correlation (*r* = 0.9889) was observed (Fig. 4).

### Relationship between pLDH units and total parasite bio-burden in mice infected with *Plasmodium berghei* ANKA luciferase

**Fig. 5:**
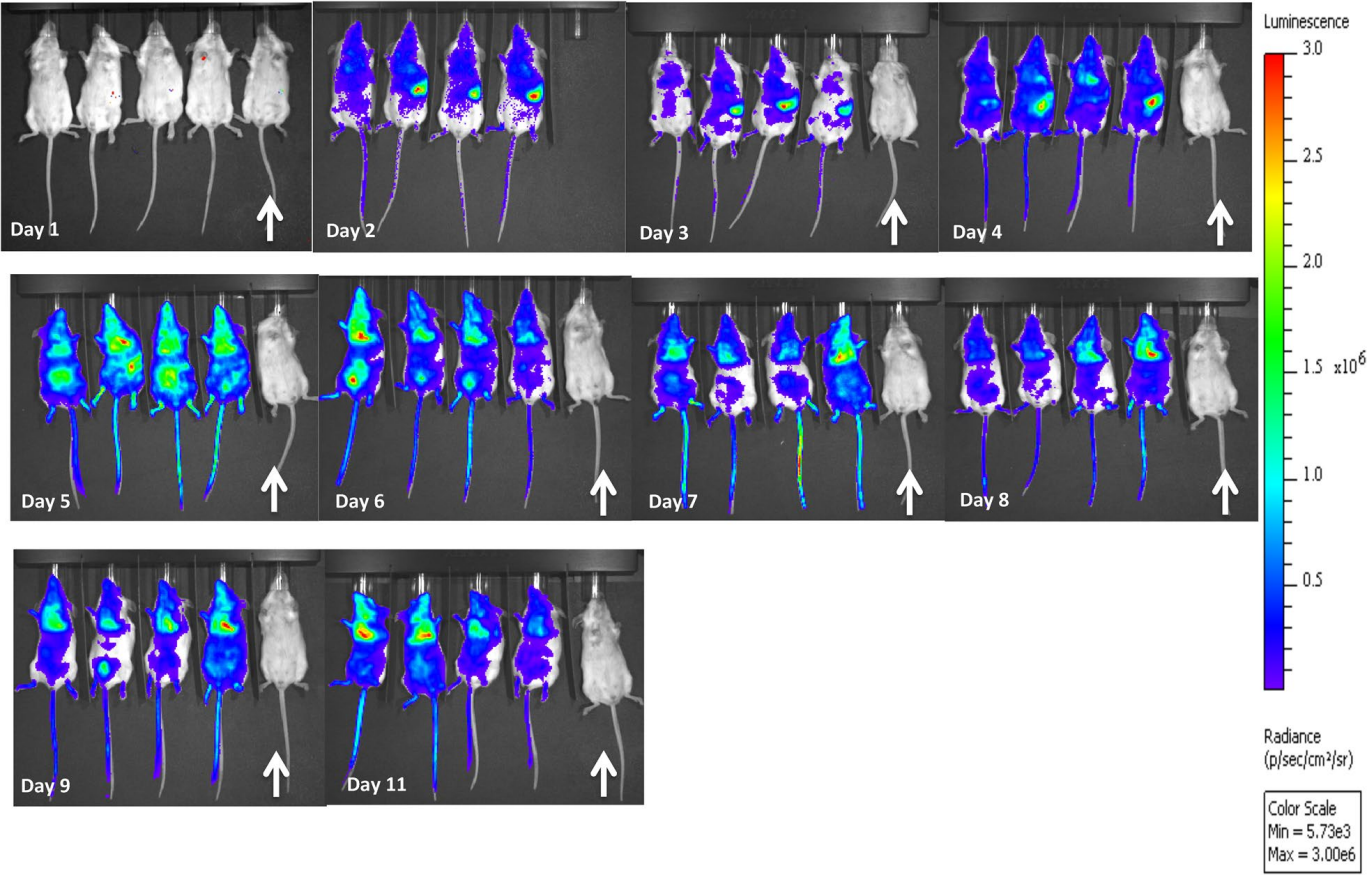
Visualization of luciferase-expressing *Plasmodium* *berghei* ANKA parasites in representative BALB/c mice. The distribution of parasites was visualized by measuring luciferase activity in animals by using an I-CCD video camera. *Rainbow images* show the relative level of luciferase activity ranging from low (*blue*), to medium (*green*), to high (*yellow*, *red*). The scale of total photon counts can be different between separate illustrations. The *white arrows* indicate a naïve mouse

**Fig. 6:**
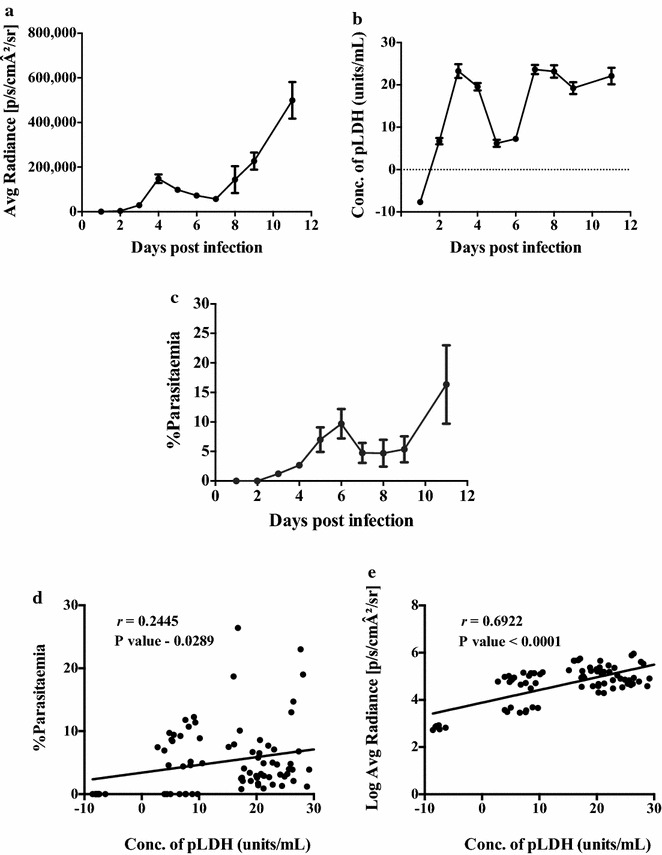
Relationship between pLDH units and total parasite bio-burden in mice (n = 16) infected with *Plasmodium* *berghei* ANKA luciferase. **a** Bio-luminescence measured as average radiance [p/s/cmA2/sr] plotted on a logarithmic scale *vs* days post infection. **b** Mean concentration of pLDH units in whole blood obtained from infected mice days post infection. **c** Mean peripheral blood parasitaemia curves in infected mice as determined by microscopy. **d** Correlation between the pLDH units and mean parasitaemia, showing the coefficient correlation (*r* = 0.2445). **e** Correlation between the pLDH units and log average radiance, showing the coefficient correlation (*r* = 0.6992). *Error bars* represent mean ± SE of biological replicates. At day 1, the OD values for the infected mice were less than the control (naïve mouse blood)

To evaluate the assay as a measure of total parasite bio-burden, BALB/c mice (n = 16) were infected with *P*. *berghei* ANKA luciferase and parasitized whole blood was collected every alternate day from eight mice post visualization under the bio-illuminator (see “Methods”) (Fig. 5). Total radiance (Fig. 6a) was determined for each mouse throughout the infection. The concentration of pLDH units and parasitaemia were also determined (Fig. 6b, c). No correlation (*r* = 0.2445) was observed between parasitaemia and pLDH units (Fig. 6d). However, a strong correlation (*r* = 0.6922) was observed between the levels of pLDH units and log average radiance (Fig. 6e).

## Discussion

Current methods for the quantification of parasite levels in blood include microscopy, qPCR and flow cytometry, which are limited to detecting circulating parasites. These assays can be expensive and are unable to quantify total parasite bio-burden. Sequestration of parasites in the cerebral regions, placenta and bone marrow leads to an underestimation of total parasite bio-burden when using these assays [47–49]. This study demonstrated that pLDH units correlate with total parasite bio-burden in murine malaria models. A human *Plasmodium* antibody-based assay was used to quantify levels of pLDH in mice infected with different murine *Plasmodium*, including species that sequester in peripheral tissues or spleen, and established this assay as a measure of total parasite bio-burden.

This study investigated the use of a commercial human malaria ELISA kit to detect levels of pLDH units in the blood and serum of infected mice. Some differences are observed between *P.falciparum* and rodent malaria LDH sequences (see above); therefore, pLDH ‘units’ rather than amount of pLDH per se has been referred to when assessing rodent parasites, with one unit being defined as the OD equivalent of 1 ng of *Pf* LDH. pLDH units were detected at lower levels in *P. vinckei*-infected blood compared to mice infected with other parasites. The assay was more sensitive for *P. berghei* ANKA, which could be due to less sequence variation in the region of the antibody-binding site compared to other parasites. However, as the binding site of the antibody is currently unknown, this could not be confirmed.

This study compared the levels of pLDH units to an established bio-burden assay with luciferase transgenic parasites [8, 13] to establish an economical, high throughput in vivo parasite bio-burden assay that is not reliant on the use of luminescent parasites or expensive equipment. A strong correlation was observed between the bio-luminescence and pLDH assays. It was of interest that a strong correlation between *P. berghei* peripheral parasitaemia and pLDH levels was not detected, which may be due to the presence of sequestered parasites as shown in the bio-luminescent images (Fig. 5). The strong correlation that was observed between *P. chabaudi* peripheral parasitaemia and pLDH levels (Fig. 4c) likely reflects the fact that this parasite is highly synchronous and blood samples were taken in the morning when the parasites are at the ring stage and in the periphery.

## Conclusions

This study demonstrates the use of an established human malaria pLDH kit for detecting murine malaria parasite bio-burden. pLDH was detected in four strains of murine *Plasmodium*. This assay permitted the estimation of pLDH in different components of infected blood, thus, accounting for both circulating and sequestered parasites. This is confirmed by the strong correlation between the bio-luminescence and pLDH units during the initial parasite growth. Therefore, this assay provides an accurate assessment of parasite bio-burden in rodent malaria species. Furthermore, these results suggest that the pLDH assay can provide a valid estimate of total parasite bio-burden in malaria-infected patients.
